# LET measurements in proton and helium‐ion beams of therapeutic energies using a silicon pixel detector towards a tool for quality assurance

**DOI:** 10.1002/mp.18085

**Published:** 2025-09-04

**Authors:** Yasmin Hamad, Ferisya Kusuma Sari, Renato Félix‐Bautista, Mária Martišíková, Andrea Mairani, Tim Gehrke

**Affiliations:** ^1^ Heidelberg Institute for Radiation Oncology (HIRO) National Center for Research in Radiation Oncology (NCRO) Heidelberg Germany; ^2^ Department of Medical Physics in Radiation Oncology German Cancer Research Center (DKFZ) Heidelberg Germany; ^3^ Faculty of Physics and Astronomy Heidelberg University Heidelberg Germany; ^4^ Heidelberg Ion‐Beam Therapy Center (HIT) Department of Radiation Oncology Heidelberg University Hospital Heidelberg Germany; ^5^ Medical Faculty Mannheim Heidelberg University Mannheim Germany; ^6^ Department of Radiation Oncology Heidelberg University Hospital Heidelberg Germany; ^7^ Present address: Currently at CH Kilger Anwaltspartnerschaft mbB 10719 Berlin Germany

**Keywords:** helium‐beam radiotherapy, linear energy transfer, Monte Carlo simulations, proton therapy, relative biological effectiveness, silicon pixel Timepix3 detector

## Abstract

**Background:**

As advanced treatment plans increasingly include optimizing both dose and linear energy transfer (LET), there is a growing demand for tools to measure LET in clinical settings. Although various detection systems have been investigated in this pursuit, the scarcity of detectors capable of providing per‐ion data for a fast and streamlined verification of LET distributions remains an issue. Silicon pixel detector technology bridges this gap by enabling rapid tracking of single‐ion energy deposition.

**Purpose:**

This study proposes a methodology for assessing LET and relative biological effectiveness (RBE) in mixed radiation fields produced by clinical proton and helium ion beams, using a hybrid silicon pixel detector equipped with a Timepix3 chip.

**Methods:**

The Timepix3 detector was placed behind PMMA slabs of different thicknesses and exposed to initially monoenergetic proton and helium‐ion beams. The detector featured a 300 µm‐thick silicon sensor operated in partial depletion. Silicon‐based LET spectra were derived from single‐ion deposited energy across the sensor and subsequently converted to water‐equivalent spectra. Track‐ and dose‐averaged LET (*LET_t_* and *LET_d_*) were calculated from these spectra. LET measurements were used as input to estimate the RBE via the modified microdosimetric kinetic model (mMKM) assuming an (α/β)*_γ_* value of 2 Gy. Measurements were compared with simulations performed using the FLUKA Monte Carlo code. Energy deposition spectra, *LET_t_* and *LET_d_* values were simulated at various depths in PMMA for the radiation fields used, by considering the contribution from the secondary particles generated in the ion interaction processes as well.

**Results:**

Energy deposition spectra were validated against Monte Carlo simulations, showing good agreement in both spectral shapes and positions. However, a depth uncertainty of less than 1 mm and other potential differences between measurements and simulations led to deviations, particularly in the distal region of the Bragg curve. Relative differences of *LET_d_* between measurements and simulations were within 3% for protons and 10% for helium ions upstream of the Bragg curves. Notably, larger discrepancies were observed in the distal part of the Bragg curve, with maximum relative differences of 7% for protons and 17% for helium ions. Average differences between RBE predictions from measured and simulated LET spectra were within 1% and 6% for protons and helium, respectively. Nevertheless, for both particle types, most measurements agreed with simulations within 1σ experimental uncertainty across the measured depths, with deviations beyond 1σ generally remaining within 3σ.

**Conclusions:**

This study demonstrates the performance of silicon pixel detectors with respect to LET measurements and RBE estimation in clinical proton and helium‐ion beams. The streamlined and accessible outline of the proposed methodology supports easy implementation into clinical routines, promising a viable and sound quality assurance tool for particle therapy.

## 1 INTRODUCTION

Proton therapy offers several advantages over conventional photon treatments, particularly for target volumes close to an organ at risk (OAR). Next to protons, helium ions are increasingly considered as a promising alternative due to their advantageous physical and biological properties.^1^, ^2^, ^3^ Compared to clinical proton beams, helium ions exhibit an improved dose conformity and a superior sparing of OARs. Furthermore, in the frame of heavy ion therapy, their reduced fragmentation tail compared to carbon ions allows to deliver a lower dose to tissues distal to the target. From a radiobiological perspective, helium ions exhibit a higher relative biological effectiveness (RBE) than protons.^3^ These features hold great potential to improve the therapeutic gain of helium ions in comparison to proton therapy making them a compelling choice in the treatment of deep‐seated solid tumors. In the light of these advantages, at the Heidelberg Ion‐Beam Therapy (HIT) center there is an ongoing effort to integrate helium ion therapy into clinical practice, with the first patients having already received treatment. In order to support the clinical application of helium ions and exploit their potential advantages, treatment planning calculations of both dose and RBE‐weighted dose distributions are necessary. In proton therapy, a constant RBE of 1.1 is applied clinically,^4^ although RBE varies with dose, linear energy transfer (LET), cell line, and chosen endpoint.^5^ For helium ion beam therapy, a variable RBE approach has been proposed.^6^, ^7^ These models are parametrized in terms of the LET, which has historically been the radiation quality specifier used to relate physical parameters and biological effects of a radiation field.

Thus, in principle, the ability to measure the LET would enable the experimental measurement of the RBE for clinical scenarios. Moreover, by obtaining LET spectra, it is possible to optimize treatments in terms of LET objectives and constraints. Studies have shown that LET painting is an effective strategy for enhancing biological effects within the tumor target while minimizing high LET components in the OARs.^8^, ^9^

Current LET optimization relies exclusively on Monte Carlo (MC) simulations, which require further validation through experimental data. High spatial and temporal resolution measurements of single‐ion LET are crucial for developing treatment‐planning methods with the goal of avoiding LET hotspots in OARs. With the growing interest in incorporating LET as an additional clinical metric in treatment planning, there is an increased need for a device capable of accurately assessing LET. This lack of standardization is in contrast to absorbed dose, for which standard devices and protocols are in place to ensure traceability and facilitate the comparison of clinical findings.^10^

Although passive detectors such as optically stimulated luminescence detectors (OSLDs), thermoluminescent detectors (TLDs), and fluorescent nuclear track detectors (FNTDs) are suitable for LET measurements, a commercially available active detector with high granularity and temporal resolution could serve as a promising and easy‐to‐use complement to other investigated methods. FNTDs suffer from time‐consuming readouts and require lower fluences than those used clinically to avoid overlapping tracks. TLDs and OSLDs integrate signals over the detection volume, preventing spectroscopic analysis. Despite their disadvantage in terms of conducting spectroscopic analysis, passive detection systems remain valuable for applications without time constraints, such as research studies, annual quality assurance, or commissioning; however, active detectors are essential for their real‐time measurement capabilities in envisaged routine clinical applications. Therefore, fast, user‐friendly ion detection devices with a high spatial and temporal resolution are of great interest. In this regard, the capabilities of Timepix3 detectors are exploited to measure the energy deposition of ions and subsequently LET spectra. Nevertheless, measuring LET spectra of single ions with Timepix3 detectors currently requires a fluence rate below clinical settings of ∼10^5^ ions s^−1^ cm^−2^ that results in a dose rate of ∼ 3–30 µGy s^−1^.^11^

This work aims at developing a methodology for experimentally assessing the LET spectra and RBE predictions in clinical proton and helium‐ion beams using a hybrid semiconductor pixel Timepix3 detector. LET spectra, track‐ and dose‐averaged LET (*LET_t_* and *LET_d_*) values were compared with MC simulations. While this work focuses on evaluating the agreement and deviations between measurements and simulations, future work will investigate the underlying causes of these differences with the aim of optimizing measurements and simulations towards quality assurance (QA) tools for assessing LET distributions in clinical settings.

## 2 MATERIALS AND METHODS

### 2.1 AdvaPIX‐Timepix3 detector

A single AdvaPIX TPX3 detector (purchased from ADVACAM, Prague, Czech Republic) was employed in this study. The detector features a 296 µm thick silicon sensor (details in reference^12^) with a sensitive area of 1.98 cm^2^, bump‐bonded to a readout Timepix3 chip with 256 × 256 pixels of 55 µm pitch. This chip was developed at CERN within the Medipix3 collaboration.^13^ The detector allows a 48‐bit‐data driven readout per event in the simultaneous time‐of‐arrival (ToA) and time‐over‐threshold (ToT) acquisition. The single‐pixel information can then be used to measure the deposited energy via ToT and time of arrival of single ions, which form clusters of hit pixels as they pass through the detector. To compensate for pixel‐to‐pixel threshold variations and ensure noise‐free data acquisition, a 3 keV energy threshold for each pixel was individually adjusted using a 4‐bit digital‐to‐analog converter. This procedure, known as threshold equalization, is described in Poikela.^13^ Energy‐deposition data recorded by Timepix3 detectors are given in a wide dynamic range together with high signal‐to‐noise ratio, which enhances the discrimination of different particle types and potential background. The detector was controlled via the PIXET Pro software, which facilitates online visualization, data readout, and pre‐processing through a USB‐based interface.^14^ The readout interface provides power and bias voltage to the sensor and controls the data acquisition.

### 2.2 Measurements

**FIGURE 1:**
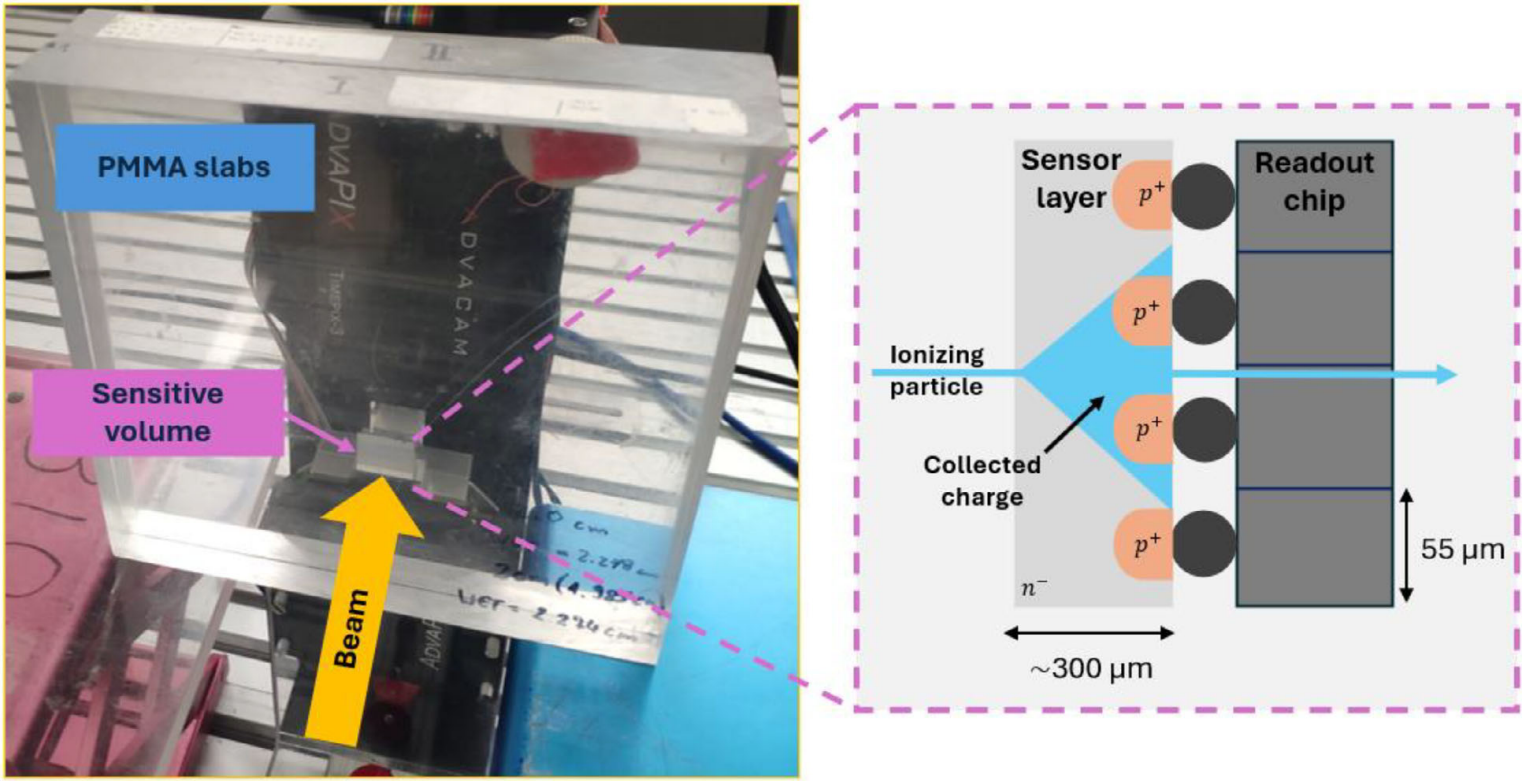
Photo of the experimental setup at HIT. The detector is placed behind PMMA slabs and the schematic view represents the sensitive silicon‐layer, where the particle charge is spread over several pixels.

Experiments were conducted in the experimental room of the HIT center in Germany.^15^ The Timepix3 detector was aligned to the isocenter using crosshair lasers, and measurements were performed downstream of polymethyl methacrylate (PMMA) slabs with perpendicular irradiation to the detector surface (cf. Figure 1). The detector was operated in a partially depleted mode with 10 V bias voltage, corresponding to a 137 µm ± 3 µm partially depleted thickness. The uncertainty corresponds to the standard deviation of four measurements over a duration of four months, which shows a good long‐term stability of ∼2%. Measurements were taken for a 148.21 MeV/u proton beam and a 149.02 MeV/u helium‐ion beam along the beam axis at different PMMA depths. The corresponding *R_90,distal_* in PMMA was 13.2 cm for protons and 13.3 cm for helium, with spot sizes (FWHM) of 13.7 mm and 11.1 mm, respectively. Energy deposition spectra were collected at depths of 42 mm, 85 mm, 120 mm, 130 mm, 131 mm, 132 mm, 135 and 150 mm for both beams. Additional helium spectra were collected free in air and at depths of 105 mm, 125 and 133 mm. The respective water‐equivalent thicknesses can be derived by multiplying it with a relative stopping power (RSP) of 1.158 ± 0.008. The RSP represents the mean and corresponding standard deviation of the five thickest used PMMA slabs as measured in range pullback measurements using the PTW PeakFinder water column.

To minimize pile‐up effects and ensure accurate detection of individual ion events, the ion‐beam intensity was reduced by two to three orders of magnitude compared with respect to the lowest clinical intensity (i.e. 10^6^–10^7^ ions s^−1^), achieving a rate of 10⁴–10⁵ ions/s. Furthermore, an air cooling system was employed to stabilize the detector temperature at around 26^°^C and prevent energy threshold drifts caused by thermal fluctuations.^16^

### 2.3 Monte Carlo simulations

MC simulations were conducted using the fully integrated particle physics simulation package FLUKA (version 2021.2).^17^, ^18^, ^19^ The predefined physics settings for precision calculations were used by means of the PRECISIOn option in the DEFAULTS command. The particle transport and the delta ray production thresholds were set equal to 10 keV. The COALESCE card was used to take into account the coalescence mechanism, a process in which high‐energy light fragments can be produced by a mechanism that brings together nucleons that are proximate in phase space. The evaporation of heavy fragments was considered by activation of the EVAPORATION card. The simulation of those processes is required to achieve accurate results on the fragments production. Moreover, for an accurate description of the transport and interactions of the beam with the traversed materials, the full beamline geometry of HIT has been included.^20^ A detailed MC model of the Timepix3 detector was implemented, encompassing all pertinent components essential for energy deposition scoring, without accounting for electronics‐related effects.^12^ Further details can be found in Table S2. In order to obtain sufficient statistics, in the form of “hits” or counts in the detector, the number of primary ions generated in the simulation was varied depending on where along the beam, i.e. along the different depths of PMMA, the detector was positioned. For positions upstream of the Bragg Peak (BP), simulations were performed with 10^7^ primary ions, while for positions downstream of the BP, due to the loss of primary beam, 10^9^ primary ions were generated.

The energy deposition spectra were scored using a customized version of the MGDRAW routine. These spectra were compared with the measured energy deposition spectra, and both simulated and experimental data were subsequently analyzed to derive *LET_t_* and *LET_d_* values, following the workflow described in the section 2.6. This approach facilitates a direct comparison between the experimental and simulated results at a specific target depth.

### 2.4 Processing of measured data

Analysis of the raw data and the identification of the particle species measured behind the target were performed using self‐written routines in C++ and MATLAB (version 9.10.0 R2021a. The MathWorks Inc., Natick, Massachusetts, USA). In the following sections, the data analysis procedure is described in detail.

#### 2.4.1 Acquisition and analysis of the particle signals in the detector

When a particle interacts with the silicon sensor, neighboring pixels around the interaction point can exhibit non‐zero signals. This group of adjacent non‐zero pixels is referred to as a cluster. Clusters are formed due to diffusion and electric repulsion of electron‐hole pairs created along the particle's path, which leads to a spread in charge collection across several neighboring pixels.^21^ These processes occur during the signal collection and registration stages.^22^ Each cluster of neighboring non‐zero signals corresponds to an ionizing particle. The cluster features and shape depend on factors such as the particle type, energy, incident angle and the applied bias voltage. A key property of a cluster is its size, given by the number of pixels in a cluster. The energy deposition information is inferred from the cluster volume, calculated as the sum of the individual keV values within a single cluster.^23^ To convert ToT values into energy deposition, a per‐pixel calibration was performed beforehand following a protocol based on X‐ray fluorescence, as detailed in Jakubek.^24^ The forming of clusters of adjacent non‐zero signals from the measured raw data is described elsewhere,^25^, ^26^ whereas the maximum allowed time difference between neighboring pixels was modified to 850 ns for the specific Timepix3 detector of this work. Cropped clusters due to erroneous time stamps assignments were identified and corrected in the same algorithm. Subsequently, energy deposition distributions were recalibrated to address saturation effects mainly caused by the so‐called volcano‐effect. The recalibration followed the relationship:

(1) $${\left\langle E_{dep}^{rec}\right\rangle }_{i}=-\frac{1}{k}ln\frac{p-{\left\langle E_{dep}\right\rangle }_{i}}{q}$$

with k = 0.155 ± 0.007, p = 6.39 ± 0.25, q = 6.40 ± 0.25. Further details about the data post‐processing can be found in Félix‐Bautista.^12^ A low‐energy cutoff around 30 keV—stemming from both the per‐pixel energy threshold and the rejection of unwanted noise in the form of single‐pixel signals—was applied, truncating the spectra at low energies.

The current post‐processing completed in less than one hour on a single core of an Intel® Core™ i5‐9500 CPU workstation. Optimized acquisition settings and a streamlined, parallelized processing pipeline might enable the collection and post‐processing of sufficient statistics within minutes.

#### 2.4.2 Ion spectroscopy

Ion type discrimination was achieved by analyzing two key cluster parameters: cluster size and cluster volume. This method, originally developed for identifying different ion types in mixed ion fields originating from therapeutic carbon ion beams,^27^ was later adapted for helium beams^28^, ^29^, ^30^ and it is applied here. Previous studies using the Medipix2 detector, the predecessor of the Timepix detector, already demonstrated successful discrimination of low‐energy electrons, photons, protons and alpha particles through pattern recognition of the detected signal.^31^

**FIGURE 2:**
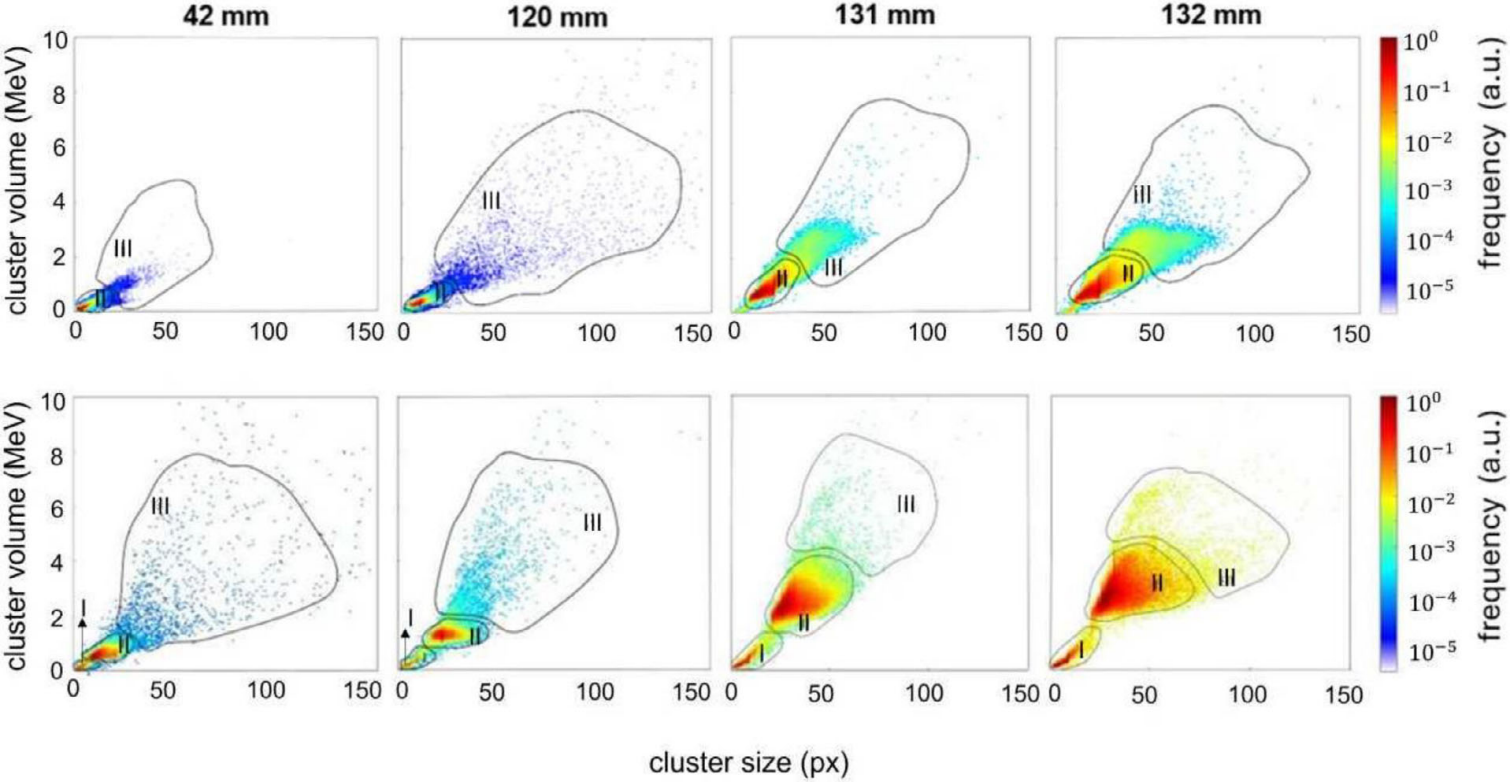
Heatmaps of all clusters for 148.21 MeV/u protons (upper panel) and 149.02 MeV/u helium ions (bottom panel) across different PMMA depths along the Bragg curve. 2D histograms of the measured clusters based on cluster size and volume parameters. The third axis displayed by a logarithmic color scale shows the number of clusters normalized to the maximum bin value. Region II includes contributions of primary ions, region I from secondary hydrogen ions due to nuclear fragmentations, and region III from events predominantly associated with target fragments.

For helium ions, the cluster size was used to distinguish them from lighter particles and detector artifacts.^32^ Moreover, since in our conditions the cluster volume is correlated with the energy deposited by the ion in the sensitive layer,^33^ it is in general higher for helium ions than for hydrogen ions. As a consequence, using both cluster size and cluster volume enhances particle type separation, as shown in the two‐dimensional (2D) scatter plots in Figure 2. To differentiate secondary particles from primary helium ions, hand‐drawn regions of different contributions based on pattern recognition in 2D histograms were used. To assess the uncertainty related to this method, smaller and larger regions were drawn and the relative differences between the under‐ and over‐estimations with respect to the reference were found to be lower than statistical uncertainties.^30^, ^34^ These regions were manually defined, with higher event densities corresponding to the primary ion species (region II), while the remaining events were attributed to secondary fragments: region I, applicable only to experiments with primary helium ions, identifies secondary hydrogen ions as projectile fragments, and region III predominantly corresponds to heavier target fragments.

### 2.5 Evaluation of energy deposition spectra

The comparison of experimental and simulated energy deposition spectra in the partially depleted volume was initially based on mean values. However, due to the Landau‐Vavilov distribution in the entrance channel, the median is considered a more robust descriptor for the skewed, right‐tailed distribution.

When comparing the overall shape of the distribution, statistical tests such as the Kolmogorov‐Smirnov or Anderson‐Darling tests were deemed unsuitable, as they can yield misleading results. These tests are overly sensitive to mismatches in peak positions, leading to larger distances between the cumulative distribution functions.

To address this challenge, a one‐dimensional gamma index (Γ) is applied to account for both the overall distribution shape and peak position shifts. The Γ‐index represents the minimum normalized Euclidean distance between points in the measured and reference distributions, ensuring that positional (Δx) and magnitude (Δy) differences are weighted relative to their respective tolerances. Mathematically, it is defined as:

$$\Gamma =min\sqrt{{\left(\frac{\Delta x}{\delta x}\right)}^{2}+{\left(\frac{\Delta y}{\delta y}\right)}^{2}}$$

where δx and δy are the user‐defined tolerance thresholds for the positional and magnitude differences, respectively. To enable a robust comparison, both experimental and simulated distributions are normalized by their areas, ensuring consistency in the counts along the y‐axis. The positional tolerance δx was set to three histogram bins, varying between 1 keV and 30 keV for protons and between 10 keV and 400 keV for helium ions, depending on the spectrum range. The magnitude tolerance δy was fixed to 3% of the normalized distributions. A high passing rate (e.g. Γ≤1) indicates good agreement between the distributions.

### 2.6 LET spectra and derivation of LET_t_ and LET_d_

LET spectra in silicon (LET_Si_) were obtained by linearly rescaling the measured and simulated energy deposition spectra by a constant track length corresponding to the partially depleted detector thickness. In this ‘straight line’ approximation, the particle tracks are assumed to be straight and parallel to the beam axis. However, this approximation has limitations, especially in the mixed‐field radiation, where notable variations in track lengths occur. Track projections (i.e., lengths) relative to the detector surface increase with depth due to scattering within the PMMA slabs.

To ensure a tissue‐equivalent translation of the spectra, LET_Si_ was converted to LET spectra in water ($\mathrm{LE}T_{H_{2}O}$). This conversion was performed using a single‐value factor based on an average silicon‐to‐water stopping power ratio of 0.541 ($\mathrm{LE}T_{H_{2}O}\;$= 0.541⋅ LET_Si_), derived from MSTAR and PSTAR using the silicon‐tissue conversion methods.^35^, ^36^ Both these approximations and limitations associated with them will be further investigated in a future work.

Typically, the radiation field incident upon an elementary volume in the material dV comprises particles of various types and energies, resulting in a distribution of LET values within dV. Rather than reporting the full particle energy spectrum, both LET_t_ and LET_d_ are commonly used to represent the particle spectrum. The corresponding expressions are given by:

$$\mathrm{LE}\;T_{t}=\frac{\sum_{i}\phi_{i}\cdot \mathrm{LE}T_{i}}{\sum_{i}\phi_{i}}\;,\mathrm{LE}\;T_{d}=\frac{\sum_{i}\phi_{i}\cdot \mathrm{LE}T_{i}^{2}}{\sum_{i}\phi_{i}\cdot \mathrm{LE}T_{i}}$$

where $\phi_{i}$ is the fluence of the charged particle *i* which has a certain *LET_i_* value. Mathematically, the *LET_t_* is the first moment of the fluence spectrum, and *LET_d_* is the second order moment of the fluence spectrum. *LET_t_* is weighted by the charged particle fluence, while *LET_d_* emphasizes the high‐LET components present in the radiation field. Both *LET_d_* and *LET_t_* are used in proton beam therapy RBE models^37^, ^38^ and in studies exploring in vivo evidence of RBE.^39^, ^40^

Several publications highlight how averaged LET values vary depending on the averaging method used.^41^ The averaging was performed considering both the full spectra of recorded events and the contribution of identified primaries (cf. section 2.4.2. Ion spectroscopy) in order to assess the relevance of secondary‐ion contributions.

### 2.7 Uncertainty calculation

The uncertainties were calculated according to the law of propagation of uncertainty, following the Guide to the expression of Uncertainty in Measurement (GUM) framework by the International Bureau of Weights and Measures (BIPM)’s GUM.^42^ Type A uncertainty was solely due to counting statistics. As radiation counting follows Poisson statistics, the number of counts N in each energy bin was associated with a standard uncertainty of $\sqrt{N}$. However, each data point typically exceeded one million detected ions, making the resulting statistical uncertainty negligible compared to other sources and was therefore not considered when assessing agreement between measurements and simulation. The simulations were performed with even higher statistics, rendering their statistical uncertainties negligible as well. Type B uncertainties were also assessed, including the recalibration of the measured energy depositions (which encompasses the uncertainty on the initial per‐pixel ToT‐to‐energy calibration based on Fe, In, and Am sources) and detector partial depletion thickness (±3 µm, potentially due to bias voltage drifts).

The former is the dominant source of uncertainty and is associated with the fit used to correct for the saturation effect known as “volcano effect”.^12^ All measured data were recalibrated using a saturating exponential fit as reported in Equation (1), and the fit parameter uncertainties were propagated into the final LET uncertainty. To account for these uncertainties, three recalibration scenarios were considered—mean, upper, and lower—which correspond to a shift along the x‐axis of the energy deposition spectra and consequently affect the LET spectra and derived quantities. As a conservative uncertainty estimation, the fit parameters were treated as positively correlated, since the variance‐covariance matrix has shown large off‐diagonal entries that cannot be neglected. Moreover, the application of a non‐linear recalibration function to single‐ion energy depositions, which was established based on measured and simulated mean energy depositions, introduces an additional mathematical systematic uncertainty. This contribution has been incorporated into the overall uncertainty budget in quadrature. Temperature‐induced shifts of energy deposition were considered negligible, as the detector is maintained at a constant temperature using an air cooling system.

### 2.8 RBE assessment

The RBE was calculated from energy spectra measurements (i.e., not relying on single averaged values) using the modified microdosimetric kinetic model (mMKM) for both protons and helium ions. Input parameters were tailored to align with the clinical experience at the HIT facility.^43^, ^44^ The calculation ensured that a biological dose of 2 Gy(RBE) was achieved for both particle types. Using tissue parameters of α*_γ_* = 0.05 Gy^−1^ and β*_γ _* = 0.025 Gy^−2^, the mMKM assumed (α/β)*_γ _* = 2 Gy. A physical dose of 1.8 Gy for protons and 1.3 Gy for helium was used for RBE assessment.

The RBE value at each measurement point was derived using the mixed beam model based on the theory of dual radiation action. The survival curve as a function of the physical dose D was obtained using the linear quadratic (LQ) parameter tables depending on LET measurements as:

$$S\left(D\right)=exp\left(-\alpha_{mix}D-\beta_{mix}D^{2}\right)$$

$$\alpha_{\mathrm{mix}}=\frac{1}{D}\;\sum_{i}\alpha_{i}\left(\mathrm{LET}\right)\cdot d_{i}=\frac{\sum_{i}\alpha_{i}\left(\mathrm{LET}\right)\cdot \phi_{i}\cdot \mathrm{LE}T_{i}}{\sum_{i}\phi_{i}\cdot \mathrm{LE}T_{i}}$$

$$\sqrt{\beta_{mix}}=\sqrt{\beta_{\gamma }}$$

where *α_i_* is the alpha LQ parameter for monochromatic radiation as a function of LET for each particle type and *d_i_* is the microscopic dose given in an infinitesimally small volume contributed to the track of a single particle *i*. Moreover, the mMKM model assigns β*_i _= *β*_γ_* such that β*_mix_* equals β*_γ_*. For mixed fields of helium ions, hydrogen and helium ion contributions were separated by applying an LET cutoff (*LET_cut_*) at the local minimum between the hydrogen and helium peaks, where tracks with LET< *LET_cut_* were treated as hydrogen ions and tracks with LET> *LET_cut_* as helium ions. All particles recorded in region III, predominantly associated with target fragments, are assumed to correspond to the primary ion species as an approximation.

## 3 RESULTS

**FIGURE 3:**
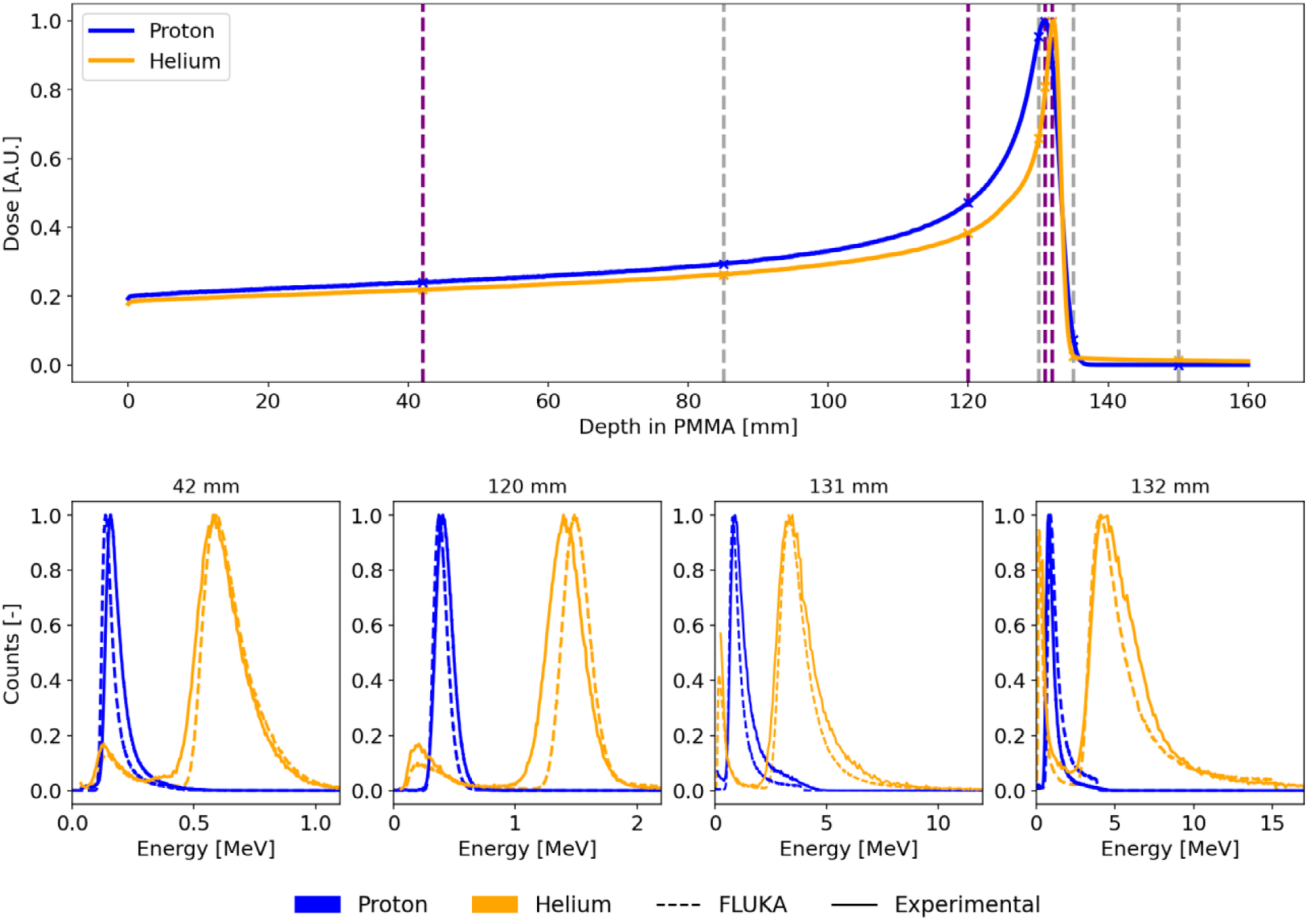
**Upper panel**: Simulated depth‐dose curves for a 148.21 MeV/u proton and 149.02 MeV/u helium beams. Dashed lines indicate the depths at which energy deposition spectra were collected in measurements and simulations, with purple dashed lines highlighting the depths shown in the bottom panel. All the spectra are reported in the supplementary section. **Bottom panel**: Comparison between simulated and experimental energy deposition spectra recorded in the partially depleted detector.

Figure 3 presents the measured and simulated energy deposition spectra at depths in PMMA of 42 mm, 120 mm, 130 mm and 131 mm for both proton and helium‐ion profiles. Data for additional depths are reported in Figures S1 and S2. The energy deposition spectra show different shapes as a function of the PMMA depth in both proton and helium‐ion fields. In addition to the shifting of the maximum energy deposition, the spectra broaden with increasing depth due to energy loss straggling. Increasing the depths the particles progressively approach the BP, where small changes in the particle energies result in large changes in LET, giving rise to broader distributions.

Measured and simulated energy deposition spectra were compared as outlined in Section 2.5. Relative differences between experimental and simulated mean and median values were calculated using the simulated values as the reference. For the helium‐ion spectra, differences are limited to a maximum of 7% for the mean and 6% for the median values upstream and at the BP. However, larger discrepancies were observed downstream of the BP, with differences rising to 20% and 35% for the median and mean values, respectively. With regard to the proton spectra, differences are not exceeding 10% in the entrance channel (42 mm–120 mm) and 5% elsewhere for the mean values. Median values showed slightly higher discrepancies, reaching up to 15% in the entrance channel and 3% elsewhere. Downstream of the BP, a pronounced increase in the median value differences was observed, reaching values up to 38%.

To evaluate the agreement between experimental and simulated normalized distributions, the 1D‐Γ index approach was applied. The gamma index computation demonstrated higher sensitivity to variations in the y‐axis threshold compared to changes in the x‐axis threshold. Differences predominantly arose from variations in relative counts on the y‐axis, rather than shifts in the position of the primary and secondary peaks. For proton spectra, gamma index passing rates remained high (above 90%) at most depths but dropped significantly at deeper regions where the proton edge—a sharp edge in the event spectrum where protons deposit their highest energy within the sensitive volume—was observed (depths 130 mm–135 mm). A 71% passing rate at 132 mm and 49.4% at 135 mm, can be attributed to a less resolved proton edge in the experimental data compared to the simulations. Conversely, helium spectra exhibited high gamma index passing rates across all depths (generally above 95%). The relative differences reported are directly transferable to LET spectra in water. This is because single rescaling factors were applied to convert energy deposition spectra into LET in silicon and subsequently to LET in water (cf. Section 2.6). Mean, median, and 1D‐gamma index passing rates values are reported in the Table S1.

### 3.1 Monoenergetic 148.21 MeV/u ^1^H beam

**FIGURE 4:**
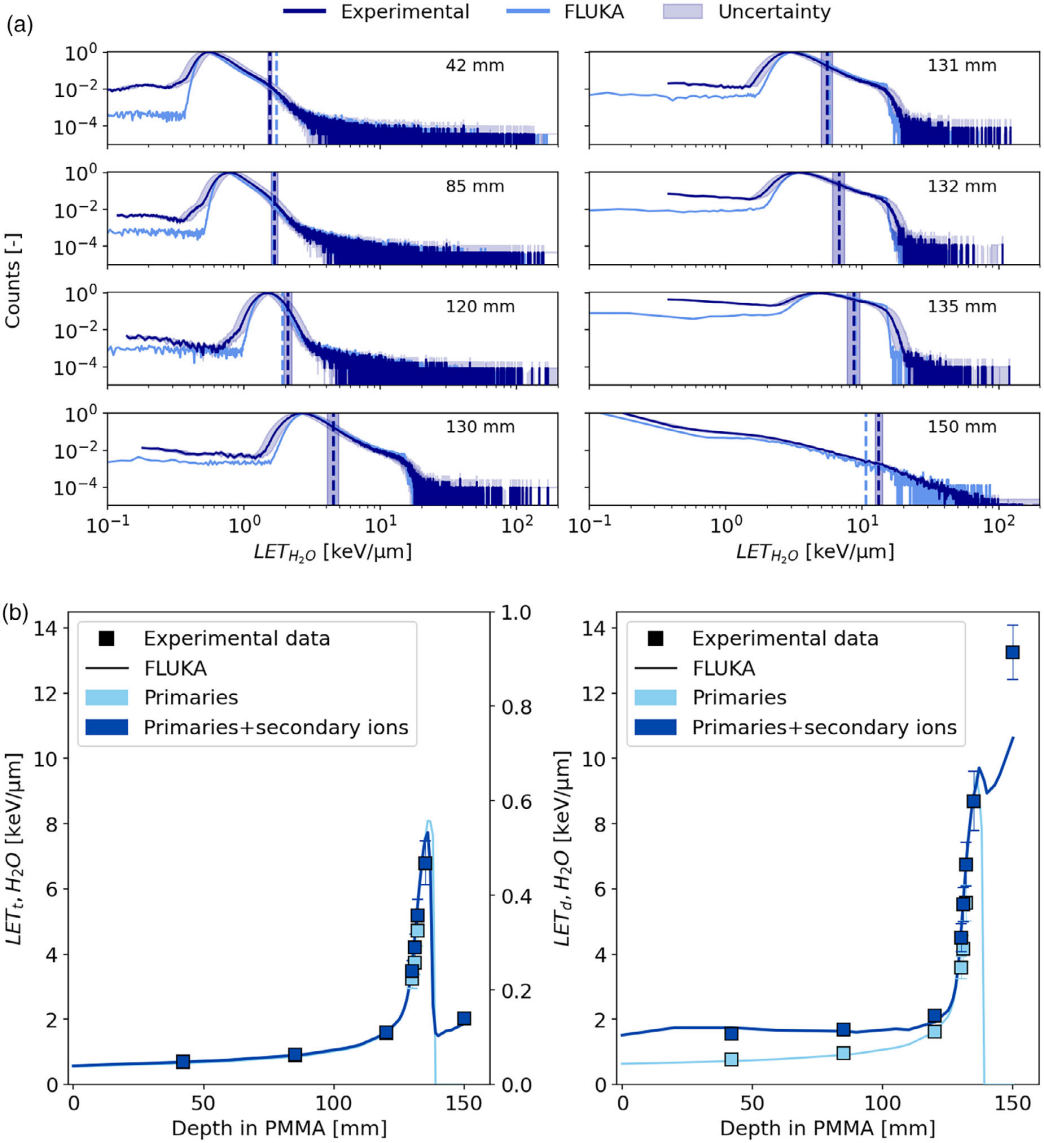
(a) Comparison between the LET spectra in water calculated with FLUKA (light blue) and the experimental measurements (dark blue) at different depths along a 148.21 MeV/u proton beam in PMMA. Vertical dashed lines on the LET spectra represent LET_d_ values obtained from measurements (dark blue) and MC simulations (light blue). The shaded uncertainty band on the experimental data reflects statistical uncertainties on the y‐axis and propagated uncertainties on the x‐axis. (b) LET_t_ and LET_d_ as a function of the depth in PMMA along the Bragg curve. Uncertainties are reported with a coverage factor k = 1.

Figure 4a presents the LET spectra in water, displayed on a log‐log scale, at various depths in PMMA along the proton Bragg curve. From the entrance channel to the BP, the $\mathrm{LE}T_{H_{2}O}$ of the ^1^H peak increases from 0.7 keV/µm to 8 keV/µm. Given the low noise level around 0.2 keV/µm, both linear and exponential extrapolations of the spectra were deemed ineffective. *LET_d_* remains relatively constant with a value of ∼2 keV/µm upstream of the BP, reaching a maximum of ∼10 keV/µm close to the BP.

The calculated track‐ and dose‐averaged LET as a function of depth are shown in Figure 4b.

When all recorded events are considered, absolute (relative) differences in *LET_d_* between experimental and simulated values remain within 0.2 keV/µm (3%) across most depths, rising to 2.6 keV/µm (7%) at 150 mm. For *LET_t_*, absolute differences are smaller, remaining below 0.5 keV/µm across all depths. When only primary particles are considered, a better agreement is observed. In this case, *LET_d_* differences are limited to 1 keV/µm (7%) across all depths, while *LET_t_* (4%) differences remain below 0.6 keV/µm. At most depths along the proton Bragg curve, experimental and simulated *LET_d_* values agree within 1σ of the experimental uncertainty, indicating very good agreement. However, in the entrance channel and beyond the BP the differences exceed 1σ but generally remain within 3σ.

### 3.2 Monoenergetic 149.02 MeV/u ^4^He ion beam

**FIGURE 5:**
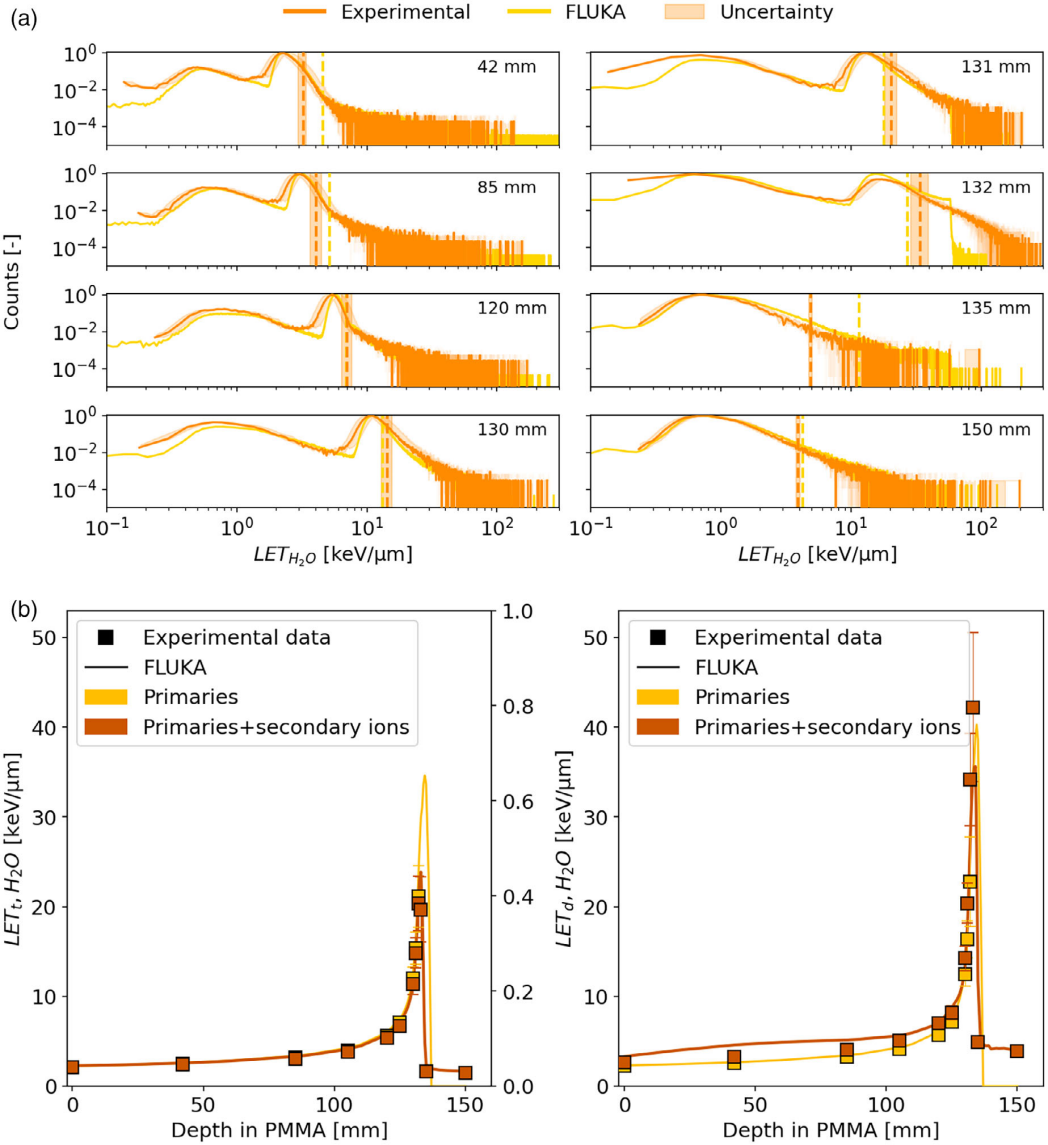
(a) Comparison between the LET spectra in water calculated with FLUKA (yellow) and the experimental measurements (dark orange) at different depths along a 149.02 MeV/u helium beam in PMMA. Vertical dashed lines on the LET spectra represent LET_d_ values obtained from measurements (dark orange) and MC simulations (yellow). The shaded uncertainty band on the experimental data reflects statistical uncertainties on the y‐axis and propagated uncertainties on the x‐axis. (b) LET_t_ and LET_d_ as a function of the depth in PMMA along the Bragg curve. Uncertainties are reported with a coverage factor k = 1.

A selection of LET spectra at different depths in PMMA when irradiated with a monoenergetic ^4^He beam is shown in Figure 5a. From the entrance channel to the BP, the $\mathrm{LE}T_{H_{2}O}$ of the ^4^He peak increases from approximately 2–11 keV/µm. The helium‐ion fragmentation increases with depth along the Bragg curve, and, correspondingly, the spectra show an increasing contribution from lighter ions. After the incident beam has stopped, the LET spectrum is significantly different as it is produced by the secondary mixed radiation field only.

In Figure 5b (right), a clear underestimation of experimental *LET_d_* values is observed when comparing results based on all detected ions, including secondaries. Despite this, absolute (relative) *LET_d_* differences are limited to 1.5 keV/µm (10%) in the entrance channel except downstream of the BP where a difference of 9 keV/µm (17%) is observed. For *LET_t_*, absolute differences are below 1 keV/µm (2%) across most depths, except around the BP where a difference of 2.66 keV/µm (3%) is noted. The observed overestimation is likely a result of uncertainties introduced by the recalibration curve applied to the experimental data and is addressed in the discussion. When considering only primaries, *LET_d_* differences do not exceed 0.6 keV/µm (6%), and *LET_t_* differences are below 0.5 keV/µm (6%), except at 132 mm depth, where a difference of 1.6 keV/µm (7%) is observed. For helium ions, agreement between simulated and experimental *LET_d_* and *LET_t_* values remains within 1σ across most measurement depths. Deviations beyond the BP and entrance channel exceed 1σ but stay within 3σ.

### 3.3 RBE prediction verification

**FIGURE 6:**
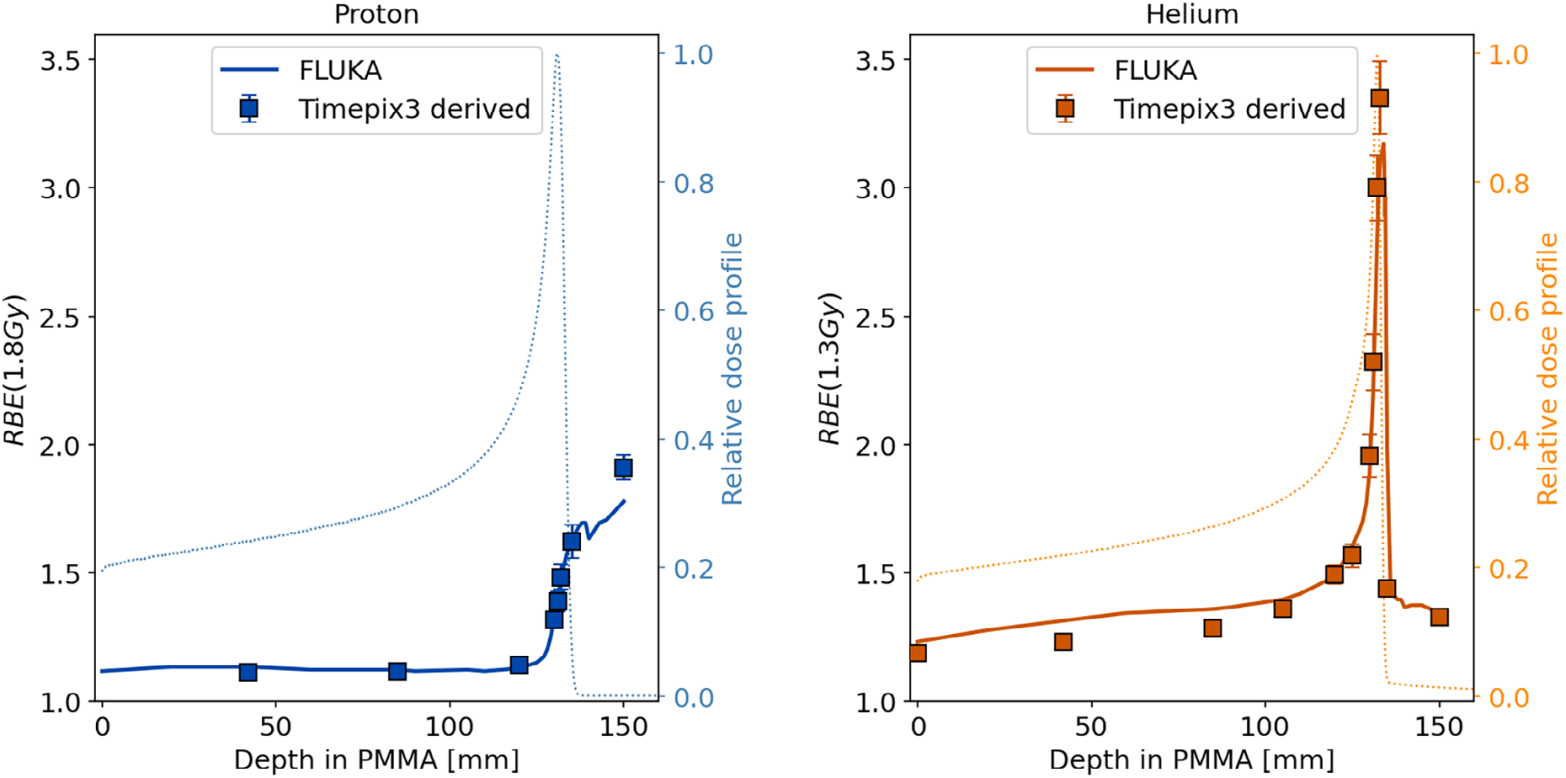
RBE predictions as a function of depth in PMMA for a 148.21 MeV/u‐proton beam (left) and a 149.02 MeV/u helium‐ion beam (right), alongside with the corresponding simulated dose distributions. Uncertainties are reported with a coverage factor k = 1.

RBE predictions of measurements and MC simulations are presented in Figure 6. A general agreement of RBE predictions is observed within 1% for protons. One exception is observed in the data point at 150 mm where a difference of 2.4% is observed. Larger RBE differences are observed for the helium data, but are still below 10% and in general measurement and simulation are in agreement within 2σ. When accounting for a positional uncertainty of ∼1 mm along the beam direction (i.e. referring to a depth uncertainty on the order of 1 mm PMMA) differences in the RBE prediction are limited to 6% along the entire Bragg curve. This same positional uncertainty affected the energy deposition spectra and, in turn, the LET values and RBE predictions. The RBE ranges from 1.11 to 1.90 for protons and from 1.18 to 3.35 for helium.

## 4 DISCUSSION

Energy‐deposition and LET spectra, averaged LET values and RBE predictions of proton and helium‐ion beams measured at various PMMA depths using the AdvaPIX detector equipped with a Timepix3 chip were compared with MC simulations (Figures 4, 5, 6). The results highlight the potential of silicon‐pixel detector technology to provide accurate LET spectra measurements, showing general agreement with MC predictions. That holds also true for data sets, in which the feature of ion identification was used to investigate the contributions of primary and secondary ions separately.

The translation of the LET spectra to RBE predictions via the mMKM model showed that the agreement between measurements and simulations even improves to deviations below 2.4% for protons and 10% for helium‐ion beams, when considering RBE along the Bragg curve.

Largest deviations were observed in the Bragg peak region, where steep LET gradients and measurement uncertainties pose the highest challenges. Observed differences between measured and simulated LET spectra may stem from interplay factors involving both the measurement method and the MC simulations. For helium ions, although not explicitly investigated, such differences could arise from limitations in the MC modeling of secondary particle production.^3^

The agreement of *LET_t_* values contrasts with the larger deviations observed for *LET_d_*, especially around the BP and distal fall‐off region. In these regions, the steep LET gradient makes measurements highly sensitive to depth uncertainties. To address this, measurements at a specific depth were compared with simulations performed at the same depth and across a range of adjacent depths. This approach enabled the assessment of depth‐related factors influencing the discrepancies, confirming the impact of positional uncertainties less than 1 mm.

Additionally, the recalibration procedure (cf. Equation (1)) introduces distortions in the energy spectra. The saturating exponential function extends the right‐tail distribution of the energy deposition spectra and amplifies the contribution of high‐LET components, where the curvature of the calibration curve is large. Such right‐tail LET contributions are weighted more heavily in the *LET_d_* calculation (cf. Section 2.6). This effect is evident in the helium case at a depth of 132 mm.

Another key source of uncertainty is the track‐length estimation. The impact of the straight‐line approximation on the LET varies depending on the energy of the particle. For high‐energy protons and helium ions, there is a minor variation of energy loss across the path length but for lower energies, particularly near the Bragg peak, the uncertainty becomes more significant. The assumption of a constant track‐length leads to an overestimation of the track‐length of ions that stop within the active volume. It leads to an underestimation of the single‐ion LET. While simulations enable the determination of the actual track length of the single event, ongoing investigations based on cluster morphology aim to overcome this intrinsic limitation on the measurements.^45^ Additional limitations, such as noise in the form of single‐fired pixels, may influence the accuracy of low‐energy deposition measurements, particularly for protons in the entrance channel or for secondary hydrogen radiation contributing to the helium spectra (cf. Figure 4a and b).

Differences in *LET_d_* may also arise from differences in the relative number of recorded charged particles^46^ and biases introduced by production thresholds of secondary radiation, such as that of delta‐rays. *LET_d_* is assumed to be a key input parameter for estimating RBE‐weighted dose distribution when integrating biological optimization into treatment planning system (TPS). However, the results show that *LET_d_* alone may not fully capture the complexity of LET spectra, especially near the Bragg peak. Using *LET_d_* as a single averaged LET value, that combines different beam qualities, does not represent all the polyenergetic tracks in the spectrum. This results in a loss of information, meaning that *LET_d_* is no longer a reliable predictor of RBE.^47^ This underscores the importance of characterizing the full LET spectrum for protons and, even more critically, for helium ions to enable robust LET‐based plan optimization.

Silicon pixel detectors are suitable candidates for LET spectra measurements due to their single‐ion detection capability. Silicon‐based detectors from the Timepix family have been used for measurements of LET based on energy deposition distributions of various proton energies in other recent works. Discrepancies of up to 12% were found in the comparison of *LET_t_* measurements and MC simulations using TOPAS^45^ and a mean deviation of 17% between *LET_d_* measurements and simulations using convolutional neural networks.^48^ A very recent work based on a Timepix3 detector for measuring LET spectra of individual energy layers of an intensity modulated proton therapy (IMPT) plan reported deviations as low as ∼5% between measured and MC‐simulated (code package: FRED) LET_d,H2O_.^49^ In this line of research the present work contributes by extending measurements of LET spectra to helium ions of therapeutic energies.

Moreover, the feature of ion identification based on hand‐drawn regions in 2D histograms of cluster size and volume is applied for investigating the LET_d, H2O_ of primary and secondary ions separately. To estimate the uncertainty related to this method of ion identification, smaller and larger regions had been drawn and the variations in terms of their counts were recently found to be lower than the statistical uncertainties based on Poissonian count statistics.^30^, ^34^ However, the robustness and applicability of the particle identification could be further improved by providing a higher degree of automatization and standardization, for example, by modern AI methods.^48^ Especially in the context of an envisaged tool for LET‐QA in clinical routine, that would be essential, but is beyond the scope of this work.

In addition, passive detectors such as FNTDs or OSLDs are also employed for LET measurements purposes.^50^, ^51^, ^52^, ^53^ Measurements with passive detectors are challenged by the demanding post‐processing, potentially limiting their frequent use in LET‐QA routines which require fast data acquisition and processing. Moreover, in the current state of research on measuring the radiation quality of ion beams, microdosimetry is considered as the golden standard. While the feasibility of microdosimetry using silicon‐on‐insulator (SOI) technology^54^ and diamond‐based detectors^55^ has been demonstrated, several limitations remain. These include non‐uniform charge collection within the sensitive volume,^56^ integration of all collected charge—hindering single‐ion identification—and practical challenges, such as an unwieldy electronic chain. Moreover, none of these methods have been commercialized and implemented in clinical practice.^11^ In this regard, silicon pixel detectors could step in to bridge this gap. The applicability of this approach for heavier ions will be investigated. Currently, limitations arise from saturation effects and signal distortions which are observed when the energy deposition per pixel is higher than ∼0.5 MeV in case of Timepix3.^57^ This limits the detector response in the detection of high‐LET particles with $\mathrm{LE}T_{H_{2}O}$ above 98 keV/µm at which its uncertainty exceeds 25%. The saturation effect is attributed to the chip circuit rather than to saturation effects in the sensor itself.^57^ However, developments by Wong^58^ related to an improved Timepix2 chip generation was recently shown to be applicable in carbon ion beams.^59^ The novel adaptive gain, which is also used in the newest Timepix4 generation,^60^ can overcome saturation effects, avoiding loss of energy‐deposition signal and extending the dynamic range. Another limitation of LET measurements with silicon pixel detectors might exist for ion‐beam therapy centers where a reduction of the fluence/dose rate below clinical settings is not feasible or allowed. However, there are multiple centers where measurements at reduced fluence rates are possible without further ado (e.g. HIT, Northwestern Medicine Chicago Proton Center,^61^ MedAustron,^62^ and the Trento Proton Therapy Centre,^63^ among others). At the HIT facility, the experimental capability has recently been extended from single‐beam irradiations to the delivery of complete IMPT treatment plans at reduced fluence rates using low beam intensity scanning. This development, which will be described in detail in a future work, constitutes a novel step beyond previous measurements restricted to single energy layers.

In this study, LET was assessed as the ratio of the energy deposited by a given particle type to the track length, as defined by the sensitive volume thickness, for both measurements and simulations. While the track length referenced in ICRU reports 16 and 85 refers to an infinitesimal value,^4^, ^64^ the track length considered in this work is at a level of hundreds of micrometers. Furthermore, although LET is fundamentally a deterministic quantity defined for a given material, particle type, and particle energy, as outlined in ICRU report 98,^65^ LET determined in this work is inherently stochastic and represented by a spectrum. This arises from the intrinsic stochastic nature of radiation interactions, that is, energy straggling, which causes event‐by‐event fluctuations in both energy deposition and track length.^66^

Experimental characterization of LET in water is essential for moving beyond absorbed dose measurements in clinical routines and validating MC‐based treatment planning systems. This study shows good agreement between measured and computed LET spectra and *LET_d_* values, offering a robust method for commissioning LET‐based proton and helium‐ion therapy plans. Currently, LET optimization relies entirely on MC simulations but complex scenarios including secondary heavy recoil particles with high LET need to be further investigated.^67^ Accurate single‐ion LET measurements can bridge the gap between MC simulations and clinical practice, enabling the development of advanced treatment planning methods to minimize LET hotspots in OARs.

## 5 CONCLUSION

As proton therapy continues to grow and advanced treatment strategies evolve towards the optimization of both dose and LET, there is a rising demand for tools to measure LET in clinical settings. This is also true for helium‐ion therapy, which could benefit from LET‐based optimization due to the higher RBE of helium compared to protons. However, there is currently no standard device for QA of radiation quality measurements, and QA routines rely solely on physical dose measurements. This study highlights the potential of the silicon pixel Timepix3 detector, which offers both single‐ion detection and spectral information, in contrast to other conventional detection systems that integrate deposited energy. A method for experimentally verifying LET spectra and assessing RBE in mixed radiation fields produced by therapeutic proton and helium‐ion beams with a hybrid silicon Timepix3 detector was proposed.

The LET spectra, averaged LET values and RBE values for both particle types were compared with MC simulation results, showing agreement within 1σ of the experimental uncertainty at 13 out of a total of 20 analyzed depths, and generally within 3σ across the full depth range. These results demonstrate that the user‐friendly interface of the Timepix3 renders it a suitable device for the assessment of LET measurements with fast and mostly automated data acquisition and evaluation.

## CONFLICT OF INTEREST STATEMENT

The authors have no conflicts to disclose.

## Supporting information



Supporting Information
