# Supplementary material for: LET measurements in proton and helium‐ion beams of therapeutic energies using a silicon pixel detector towards a tool for quality assurance
Source: Med Phys. 2025 Sep 4;52(9):e18085. doi: 10.1002/mp.18085 (PMC12409771; doi:10.1002/mp.18085)
Supplement: Supplementary file 1 — Supporting Information [file MP-52-0-s001.docx]

**Supplementary material**

**Parameters used for the simulations**

A detail summary of the simulation design and parameters is now provided in Table S-2, according to the recommendations drawn by the Task Group 268 of the American Association of Physicists in Medicine (AAPM)^68^.

|  | Description |
| --- | --- |
| Software | FLUKA software version 2021.2.3 |
| Hardware | Desktop with Intel Core i7-8700 CPU (6 cores, 12 threads), 3.20 GHz CPU clock rate, 16.0 GB RAM |
| Geometry | Full and detailed nozzle beamline and detector geometry (proprietary information). No ripple filter used.  Slabs: 20 cm x 20 cm x Z cm PMMA with Z variable thickness along the beam direction. Exit surface placed directly upstream of the detector geometry. Detector at isocenter, 1.20 m from nozzle exit. Space between the nozzle and phantom filled with air. |
| Materials | Materials from the FLUKA database.  PMMA defined as a custom material using reported composition and density. |
| Source | Single spot beam defined right upstream of the vacuum exit window.  Gaussian-like beam: FWHM 9.85 mm and 0.596 GeV total energy for helium; FWHM of 9.54 mm and 0.149 GeV total energy for protons. |
| Physics and Transport | Evaporation and coalescence activated.  Delta rays produced at 100 keV.  FLUKAFIX set to 0.05 for energy loss per step. |
| Scoring | Customized MGDRAW routine (Fortran code) used to assess deposited energy, track-length, particle type, generation flag, and number of generated events in volume of interest, region membership, and parent particle ID. Scoring in partially depleted silicon volume. Electrons and particles with 1 < Z < 8 scored and discriminated; Z > 8 scored as heavy fragments. |
| Analysis | LET spectra derived from MGDRAW scorer results analyzed offline. RBE predictions for protons and helium ions obtained using mMKM model. |
| Discussion | More comprehensive detector readout study requires other simulation toolkits such as Allpix2, to simulate saturation effects and charge diffusion affecting cluster size and volume. Fine tuning of density and material composition would improve simulation outcomes, especially near beam range end. |

**Table S-1:** Overall parameters and description of the simulations.

|  | Helium | | | |  | Proton | | | |  |
| --- | --- | --- | --- | --- | --- | --- | --- | --- | --- | --- |
|  | Experimental | | Simulations | |  | Experimental | | Simulations | |  |
| PMMA  [mm] | Mean [MeV] | Median  [MeV] | Mean  [Mev] | Median  [MeV] | Γ (%) | Mean  [MeV] | Median  [MeV] | Mean  [MeV] | Median  [MeV] | Γ (%) |
| 0 | 0.543±0.05 | 0.526±0.04 | 0.572 | 0.537 | 97.8 |  | | | |  |
| 42 | 0.603±0.05 | 0.601±0.05 | 0.647 | 0.625 | 96.9 | 0.178±0.02 | 0.158±0.01 | 0.176 | 0.153 | 99.2 |
| 85 | 0.776±0.07 | 0.789±0.07 | 0.830 | 0.829 | 97.0 | 0.234±0.02 | 0.216±0.02 | 0.230 | 0.207 | 98.7 |
| 105 | 0.967±0.09 | 0.993±0.09 | 1.039 | 1.057 | 97.6 |  | | | |  |
| 120 | 1.357±0.125 | 1.399±0.129 | 1.446 | 1.494 | 97.3 | 0.406±0.04 | 0.394±0.04 | 0.406 | 0.391 | 97.9 |
| 125 | 1.690±0.159 | 1.761±0.165 | 1.819 | 1.864 | 97.4 |  | | | |  |
| 130 | 2.878±0.282 | 2.901±0.284 | 2.873 | 2.935 | 96.2 | 0.876±0.08 | 0.764±0.07 | 0.953 | 0.821 | 86.2 |
| 131 | 3.755±0.377 | 3.619±0.364 | 3.682 | 3.558 | 90.2 | 1.06±0.100 | 0.895±0.08 | 1.161 | 0.972 | 83.1 |
| 132 | 5.294±0.499 | 4.824±0.493 | 5.150 | 4.694 | 94.9 | 1.303±0.122 | 1.09±0.100 | 1.387 | 1.163 | 71.0 |
| 133 | 5.08±00.437 | 4.686±0.433 | 5.954 | 5.866 | 68.1 |  | | | |  |
| 135 | 0.404±0.04 | 0.287±0.03 | 0.617 | 0.347 | 95.2 | 1.709±0.164 | 1.553±0.144 | 1.904 | 1.756 | 49.4 |
| 150 | 0.363±0.03 | 0.271±0.02 | 0.412 | 0.297 | 96.0 | 0.493±0.04 | 0.139±0.01 | 0.438 | 0.107 | 91.8 |

**Table S-2** : Mean values, medians, and gamma index passing rate results for all measured depths. The table provides a comprehensive overview of the statistical descriptors and agreement metrics across different depths.


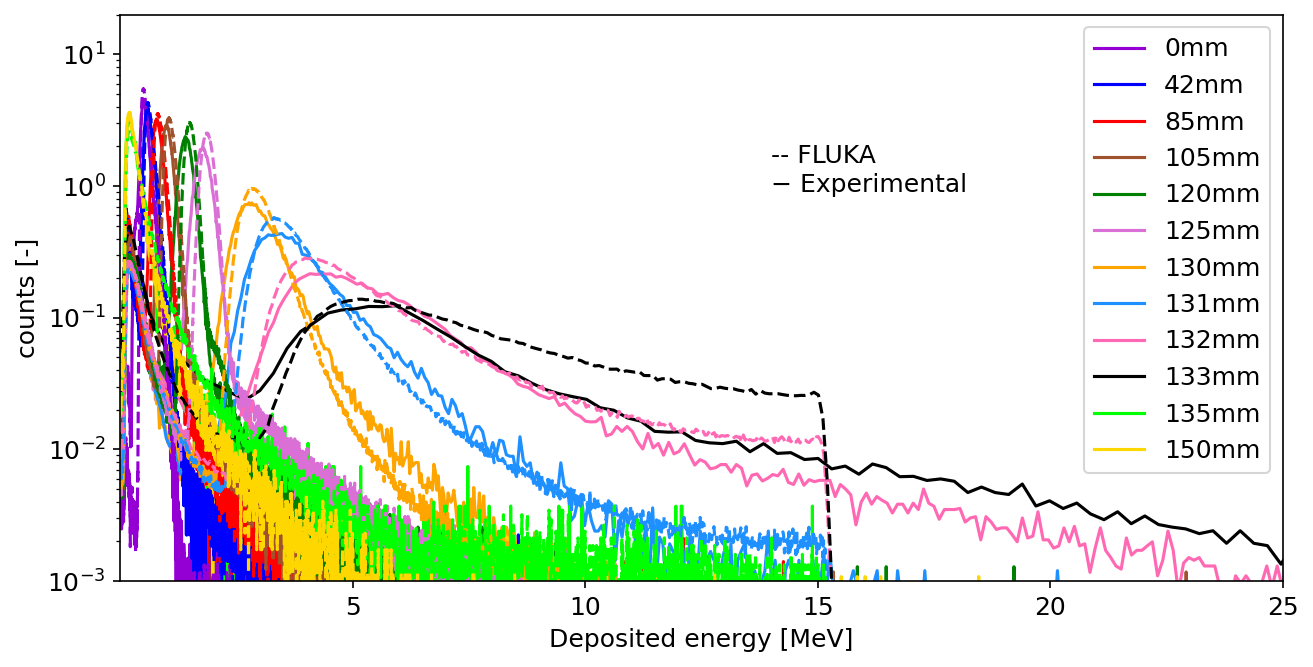


**Figure S-1*.*** *Energy deposition spectra normalized with respect to area at different depths along a 149.02 MeV/u helium Bragg curve, shown on a semi-logarithmic scale.*
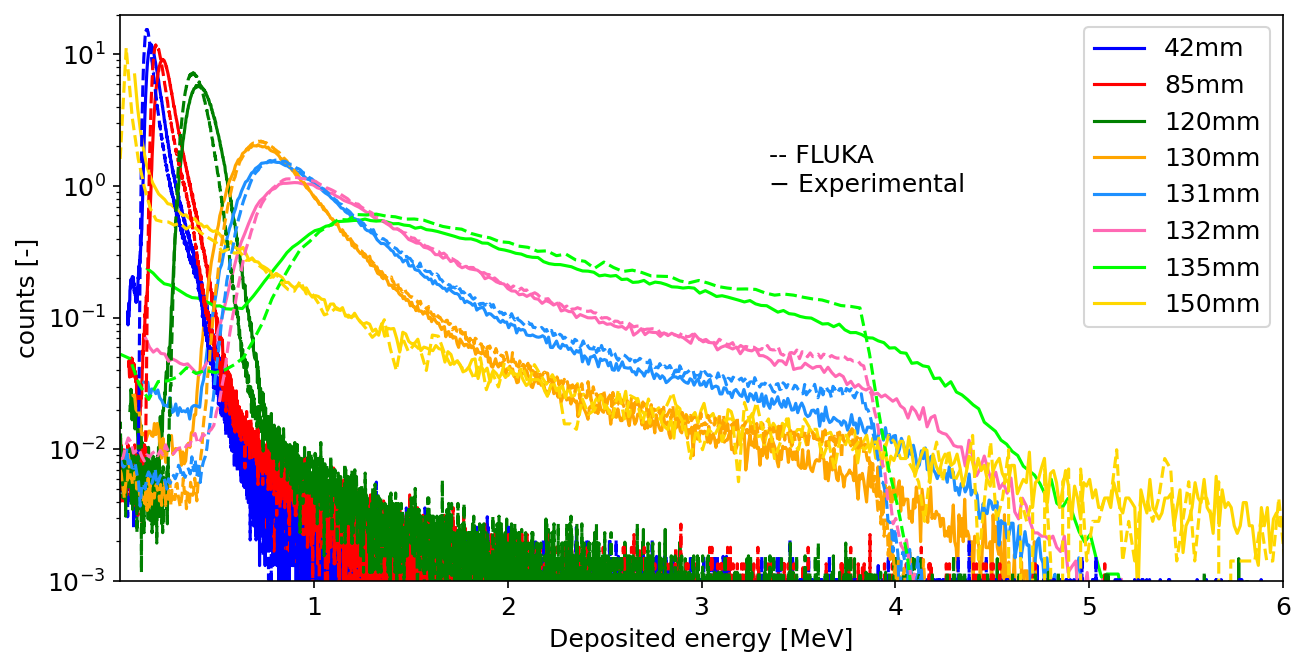


**Figure S-2.** *Energy deposition spectra normalized with respect to area at different depths along a 148.21 MeV/u proton Bragg curve, shown on a semi-logarithmic scale.*
